# In-Plane Compressive Responses of Non-Homogenous Re-Entrant Honeycombs Fabricated by Fused Deposition Modelling

**DOI:** 10.3390/mi15060694

**Published:** 2024-05-24

**Authors:** Ahmad Baroutaji, Hamid Nikkhah, Arun Arjunan, Sadjad Pirmohammad, John Robinson

**Affiliations:** 1 Faculty of Engineering and Physical Science, Aston University, Aston Triangle, Birmingham B4 7ET, UK; 2 Faculty of Engineering, School of Mechanical Engineering, University of Mohaghegh Ardabili, Ardabil 56199-11367, Iran; hamidcadd@yahoo.com (H.N.); s_pirmohammad@uma.ac.ir (S.P.); 3 School of Engineering, Faculty of Science and Engineering, University of Wolverhampton, Telford TF2 9NT, UK; a.arjunan@wlv.ac.uk (A.A.); j.robinson12@wlv.ac.uk (J.R.)

**Keywords:** auxetic structures, re-entrant honeycombs, additive manufacturing, FDM, in-plane compression

## Abstract

Auxetic structures, with re-entrant (inverted hexagonal or bow tie) unit cells, have received considerable interest due to their negative Poisson’s ratio property that results in superior mechanical properties. This study proposes a simple method to create non-homogeneous re-entrant honeycombs by modifying the size of chevron crosslinks. The various structural designs were conceived by changing the geometrical dimensions of the crosslinks, namely the length ($l_{cl}$) and the thickness ($t_{cl}$), while maintaining the parameters of the re-entrant cell walls. The influence of the design parameters of chevron crosslinks on the mechanical behaviour of additively manufactured re-entrant honeycombs was investigated experimentally and numerically. The structures were fabricated using the Fused Deposition Modelling (FDM) technique from polylactic acid (PLA) plastic. In-plane quasi-static compression tests were conducted to extract the elastic, plastic, and densification properties of the structures. Furthermore, a Finite Element (FE) model was developed via LS-DYNA R11.0 software, validated experimentally, and was then used to obtain a deeper insight into the deformation behaviour and auxetic performance of various designs. The obtained results revealed that the mechanical performance of re-entrant honeycombs can only be tuned by controlling the geometrical configuration of chevron crosslinks.

## 1. Introduction

Man-made, nature-inspired cellular structures, or metamaterials, constructed from stacking multiple unit cells, exhibit advanced mechanical performance as they combine low density with high strength and high energy absorption capacity. Therefore, these structures have attracted widespread applications in the automotive, aerospace, biomedical fields, etc. [1,2,3]. Depending on Poisson’s ratio, the cellular structures can be classified into standard and auxetic. The auxetic structures have Negative Poisson’s Ratio (NPR), meaning that they contract laterally when they are compressed axially and vice versa. The structures with NPR not only have lightweight and high-strength properties, but also demonstrate other desirable characteristics such as enhanced shear modulus, high impact and indentation resistances, and high fracture toughness [4,5,6]. Depending on the geometrical configuration of the unit cells, the auxetic structures can broadly be divided into re-entrant [7], double arrowhead (double-V) [8,9], double-U [10,11], star-shape [12,13], chiral [14,15], anti-chiral [16,17,18], and rigid rotating unit [19,20,21] structures.

**Figure 1:**
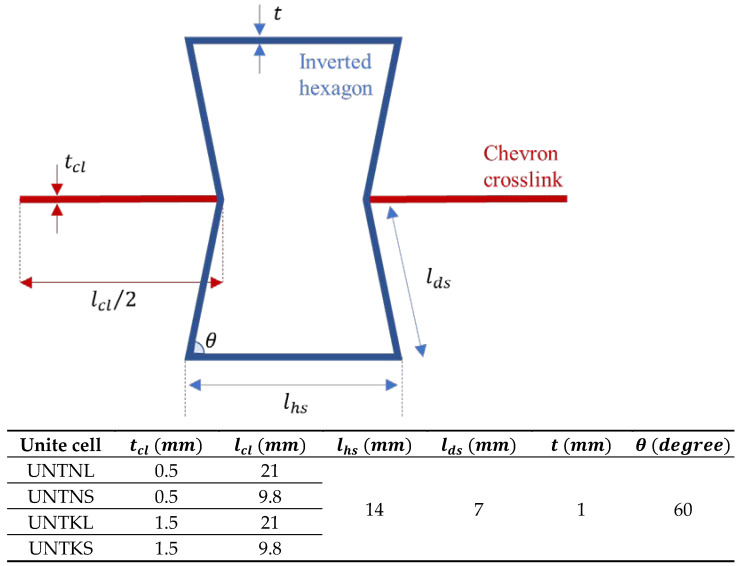
Geometrical configuration and design parameters of re-entrant unit cell.

Re-entrant honeycombs are the most researched auxetic structures due to their relatively simple unit cell design compared to other auxetic architectures. The term “re-entrant” refers to a structural arrangement that has inwardly inclined elements, i.e., elements with a negative angle, to produce the NPR effect [22]. The basic re-entrant unit cell features eight struts—six to form the inverted hexagonal shape and two crosslinks (Figure 1). The mechanical performance of re-entrant honeycombs under quasi-static and dynamic loadings was explored extensively using analytical, experimental, and numerical techniques.

Earlier research studies have focused on the elastic performance of re-entrant structures. Masters and Evans [23] developed analytical expressions for estimating the elastic properties, including tensile and shear modulus and Poisson’s ratio of re-entrant structures. They reported that the elastic behaviour of the structures can be either isotropic or anisotropic depending on the geometry of the cell. Wan et al. [24] conducted an analytical analysis based on a large deflection model and derived mathematical equations describing the strains and Poisson’s ratios of auxetic honeycombs. The study reported that geometric parameters of the re-entrant cell have a significant impact on the magnitude of the Poisson’s ratio. Dong et al. [25] explored the influence of the number of cells and wall thickness on the quasi-static compressive responses of metallic auxetic structures. They observed that the deformation mode of the structure changes significantly by changing the thickness of the cell walls. Tunay et al. [26] investigated the in-plane energy absorption performance of FDM-fabricated re-entrant structures. They found that, among the different design parameters, the thickness of the struts has the highest effect on the energy absorption metrics. Zhang et al. [27] used finite element simulations to study the influence of geometrical parameters, including cell-wall angles and edge thickness, on the in-plane dynamic crushing of auxetic honeycombs. They reported that the plateau stress and the energy absorption capacity can be enhanced by increasing the strut angle. Hu et al. [28] studied the influence of cell-wall angle on the dynamic behaviour of auxetic honeycombs. It was found that the NPR behaviour increases with decreasing cell-wall length ratio and increasing cell-wall angle. Alomarah et al. [29] examined the tensile properties of re-entrant honeycombs and found that the loading direction has a profound impact on the mechanical and auxetic responses. 

Recently, many researchers focused on modifying the geometrical configurations of the simple re-entrant unit cell and structures to form novel structures with enhanced performance. For example, Shao et al. [30] investigated the dynamic compressive performance of functionally graded auxetic re-entrant honeycomb. It was found that the grading direction, i.e., increasing or decreasing the thickness, has a significant influence on the mechanical responses of the structures. Harkati et al. [31] developed analytical models to estimate in-plane elastic constants of auxetic structures with curved re-entrant cell walls. Zhang et al. [32] modified the re-entrant honeycomb by replacing the inclined struts with an arc-shaped structure. The modified auxetic honeycombs exhibited a better energy absorption performance than the conventional honeycombs, which demonstrated a higher crushing load efficiency and lower peak stresses. Usta et al. [33] analysed the energy absorption behaviour of novel re-entrant structures composed of either asymmetric or slotted re-entrant unit cells. The study revealed that the modified structures have higher stiffness, strength, energy absorption capacity, and deformation stability than the standard re-entrant structures. Ingrole et al. [1] compared the in-plane compression behaviour of regular honeycombs, re-entrant auxetic honeycombs, locally reinforced auxetic-strut structures, and hybrid structures combining regular and auxetic honeycombs. They found that the hybrid structures provided a superior compressive strength and energy absorption than the conventional auxetic honeycombs. Alomarah et al. [34] created a hybrid auxetic structure by combining the topological features of re-entrant honeycombs and anti-tetrachiral honeycombs. The hybrid structure showed higher tensile properties and NPR effect than the standard re-entrant honeycomb. 

Due to complex geometry, it is somewhat challenging to manufacture accurate cellular structures using conventional manufacturing techniques such as investment casting, direct foaming, and stamping forming, for metals; and extrusion and injection moulding for polymers [22,35,36,37]. Therefore, Additive Manufacturing (AM) methods, with their significant design freedom and ability to fabricate parts with highly intricate shapes, become very popular to prepare auxetic and other cellular structures. AM enables the efficient and flexible production of cellular structures with novel designs from versatile materials. Many AM processes, such as Selective Laser Melting [38,39,40,41], EBM [42,43], Multi Jet Fusion (MJF) [44], and FDM [1,45,46] were used in the literature to manufacture auxetic structures from different types of materials including metals, ceramics, polymers, and composites. Among the various AM methods, FDM was attractive in the industrial context due to the low cost of printing devices and filament materials. FDM is a material extrusion process in which filament material is heated in the extruder to reach a semi-liquid state; it is then extruded through the nozzle head on a fabrication bed [47]. FMD is a sophisticated process where the quality of the printed part is governed by complex interactions between different printing and material factors [48]. The building orientation, i.e., build strategy; process parameters, such as platform temperature, nozzle temperature, printing speed, layer thickness, infill density, and raster width and angle; and properties of filament materials have a profound influence on the mechanical responses of the FDM-manufactured parts [49,50,51,52,53]. FDM-manufactured parts possess highly anisotropic mechanical properties, i.e., properties in a specific direction are different to those in other directions, due to its layer-by-layer building strategy [48]. 

From the literature survey mentioned above, it is clear that the previous work on re-entrant structures focused mainly on exploring the influence of cell geometrical parameters such as cell wall angle, thickness, length, and shape ratios (thickness/length, straight length/diagonal length) on its mechanical properties. In all these studies, the crosslink’s geometrical parameters, i.e., thickness and length, were considered identical to the parameters of the horizontal struts in the inverted hexagon shape. Therefore, the primary aim of the current paper is to isolate the parameters of crosslinks and analyse their influence on the mechanical and energy absorption responses of re-entrant structures.

## 2. Methodology

### 2.1. Re-Entrant Architecture

Re-entrant Honeycombs (RHs) with modified unit cells are considered in this study. The modification involved changing the geometrical parameters of chevron crosslinks, i.e., thickness ($t_{cl}$) and length ($l_{cl}$), resulting in four design architectures including re-entrant unit cells with thin–long (TNL), thin–short (TNS), thick–long (TKL), and thick–short (TKS) chevron crosslinks. The geometry of the basic inverted hexagonal shape was maintained in the different structures. This geometry has four parameters, including the length of the horizontal strut ($l_{hs}$), the length of the diagonal strut ($l_{ds}$), the angle of the diagonal strut (θ), and the thickness of the strut (*t*). The selected dimensions for $l_{hs}$, $l_{ds}$, θ, and *t* are 14 mm, 7 mm, 60°, and 1 mm, respectively. The unit cells with the different types of crosslinks were named UNTNL, UNTNS, UNTKL, and UNTKS. Figure 1 summarizes the geometrical parameters of the re-entrant unit cells. 

**Figure 2:**
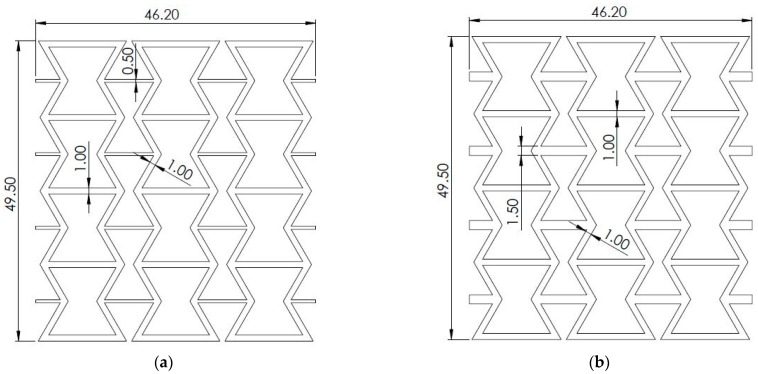

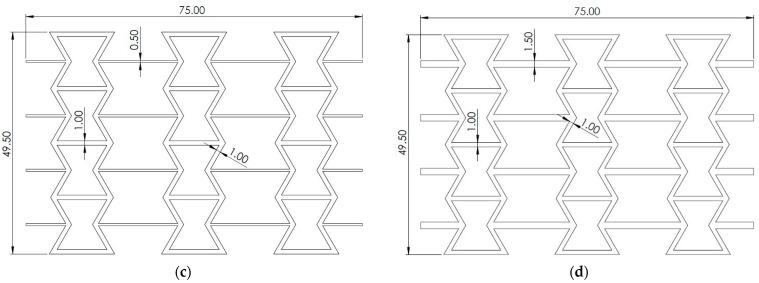
Re-entrant honeycombs: (**a**) RHTNS, (**b**) RHTKS, (**c**) RHTNL, and (**d**) RHTKL.

The re-entrant honeycombs (Figure 2) are composed of four unit cells in the vertical direction, resulting in an overall height (H) of 49.5 mm, and three unit cells in the horizontal direction, resulting in overall lengths (L) of 46.2 mm and 75 mm for structures with short and long crosslinks, respectively. All the structures have a width (W) of 10 mm. According to the configuration of the crosslink, the four different re-entrant honeycombs were named as RHTNL, RHTKL, RHTNS, and RHTKS. The RHs have different overall dimensions; therefore, their masses are different. RHTNL, RHTKL, RHTNS, and RHTKS honeycombs have masses of 7.85 g, 10.48 g, 7.123 g, and 8.3 g, respectively.

### 2.2. Materials and Manufacturing

**Figure 3:**
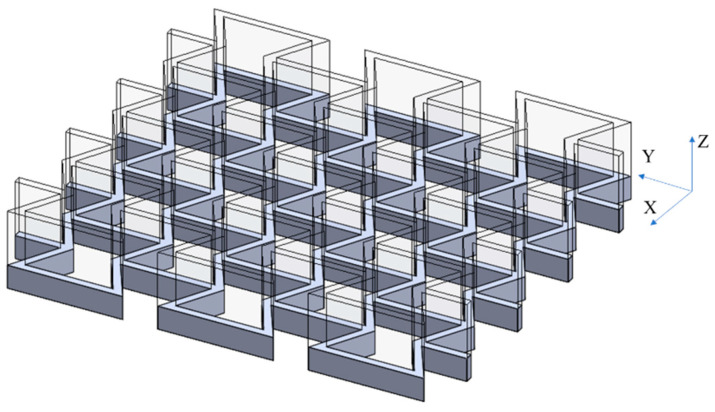
The FMD building orientation of re-entrant honeycombs.

**Figure 4:**
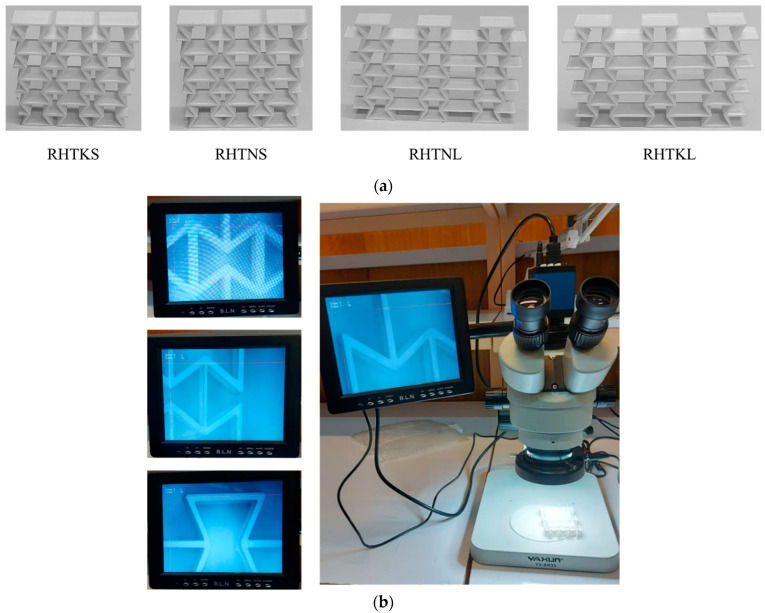
(**a**) FDM-fabricated re-entrant honeycombs. (**b**) Inspecting the quality of FDM-fabricated auxetic structures using YAXUN YX-AK33 microscope (×50 magnification).

In this work, the re-entrant honeycombs with modified unit cells were made of PLA and manufactured using FDM. Three-dimensional CAD designs of honeycombs were prepared and exported to the STL format. The STL files were then imported into the 3D printer software to convert the model into G-code files. A commercial machine (Ender 3 V2) was used for the FDM process. This 3D printer has a print precision of ±0.1 mm, a layer thickness in the range of 0.1–0.4 mm, a typical printing speed in the range of 50–60 mm/s, and a bed temperature of ≤100 ℃. The 3D printing process parameters were an extruder temperature of 220 °C, a bed temperature of 60 °C, a layer thickness of 0.2 mm, and an infill density of 100%. The zigzag infill pattern with a bidirectional raster angle was selected where the raster angles, i.e., the direction of infill lines, of successive layers were ±45°. These parameters were commonly used in similar studies [26]. The samples were built in the Z-direction, as shown in Figure 3. This building orientation was selected to avoid the need for support structures. To verify the quality of the printing process, the printed structures, shown in Figure 4a, were inspected closely using precision measuring tools and a microscope (Figure 4b). A digital calliper and micrometre were used to measure the overall dimensions, i.e., length, height, and width, at multiple locations of the printed samples. The thickness of the struts was estimated using the microscopic images. FDM-printed samples showed a high dimensional accuracy, where their dimensions were highly comparable to the design values, i.e., CAD models. Also, the visual and microscopic inspections confirmed that the fabricated structures have smooth surfaces and are free from common FDM process defects such as warping, curling, or cracking (delamination). 

### 2.3. Compression Test

**Figure 5:**
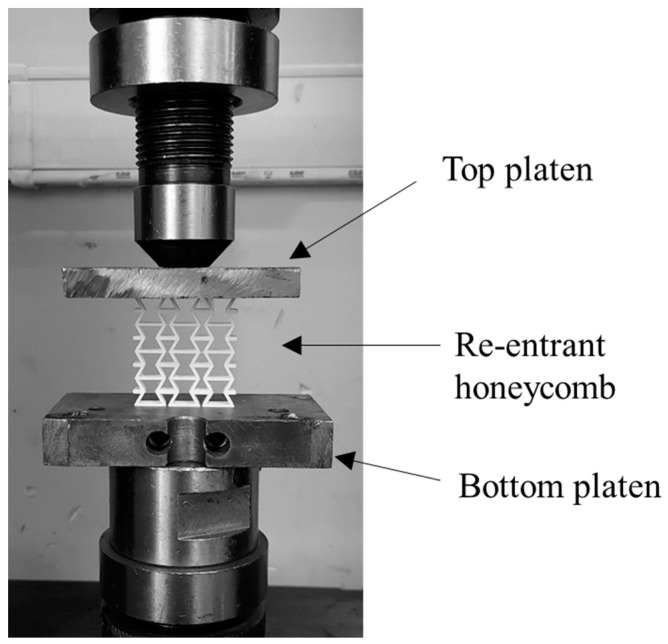
Experimental setup of the compression test using universal testing machine (STM 150 kN).

The mechanical performance of re-entrant honeycombs can be assessed by conducting compression tests. In this work, the compression tests were implemented using a Universal Testing Machine (UTM) fitted with a 150 kN load cell, as illustrated in Figure 5. The tested specimens were placed on the machine platen and aligned in a way that their top and bottom surfaces were parallel to and fully covered by the compression platens to ensure they were subjected to uniformly distributed loads during the test. The velocity of the moving platen was set to 10 mm/min to ensure a quasi-static loading scenario without triggering any dynamic effects. This velocity value is consistent with those reported in previous research studies on quasi-static compression loading [54,55]. Force and displacement datasets were recorded during the test and were then processed to produce the different mechanical responses of the tested structures. 

### 2.4. Numerical Simulation

**Figure 6:**
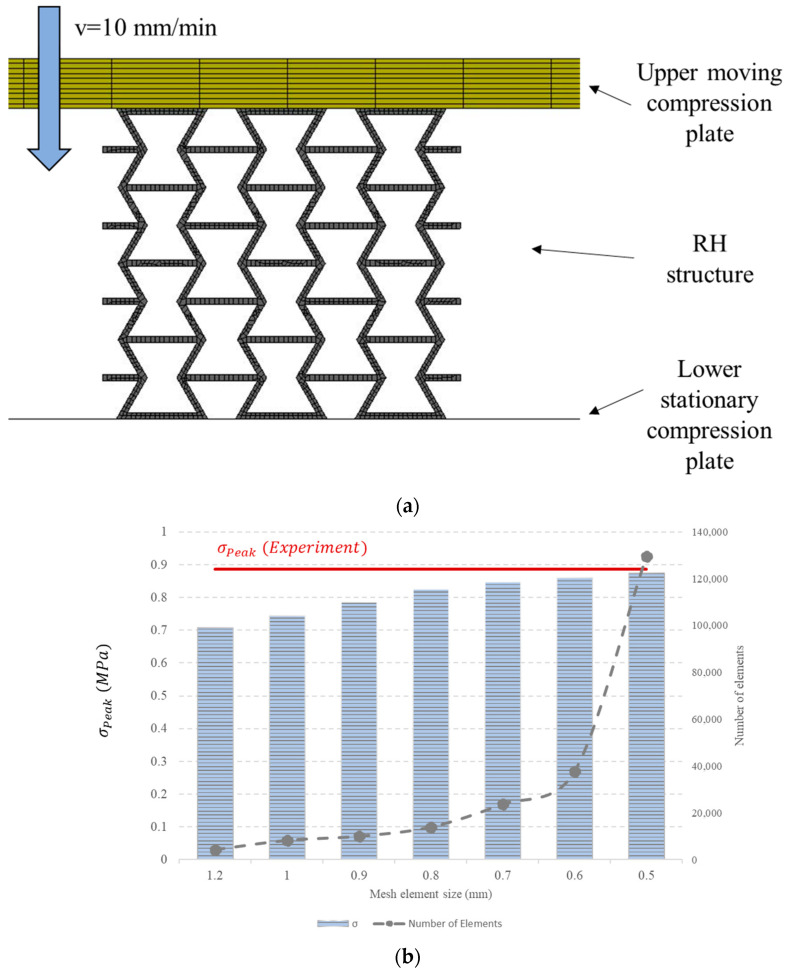
(**a**) Components and boundary conditions of the FE numerical model. (**b**) Mesh density analysis.

The Finite Element Method (FEM) was used to simulate the deformation behaviour of the auxetic honeycombs and to extract the quasi-static compressive responses. A commercial general-purpose nonlinear explicit FEM simulation software, LS-DYNA, was utilized to construct the model. Figure 6a shows the setup of the Finite Element (FE) model. The model is composed of three parts including the top and bottom platens, as well as the auxetic re-entrant honeycomb. The top and bottom platens were modelled as rigid bodies, while the RH structure was defined as a deformable part. The translation and rotational motions of the bottom platen were constrained in all *x*, *y*, and *z* directions. On the other side, the top platen was constrained to move vertically at a speed of 10 mm/min to compress the RH sample simulating the quasi-static loading condition applied in the actual experiment. The 8-node solid brick element was used to mesh the RH structure. The element size, i.e., mesh density, was determined based on a mesh convergence analysis. Seven mesh sizes (0.5 mm, 0.6 mm, 0.7 mm, 0.8 mm, 0.9 mm, 1 mm, and 1.2 mm) were used in the analysis, and the peak stress ($\sigma_{Peak}$) value was monitored. Figure 6b compares $\sigma_{Peak}$ values for the different mesh sizes. The coarse mesh, with a mesh size of 1.2 mm, had a low number of elements and required less processing time; however, its accuracy was low, as the simulated value was 20% less than the experimental one. On the other side, the finer mesh, with an element size of 0.5 mm, provided a better prediction ability with only a 1.4% error, but required a significantly greater solution time due to the increased number of elements. Therefore, an element size of 0.6 mm was selected because it provides a good balance between the accuracy and the efficiency of the model. The interactions between the different components of the model were simulated by using an automatic frictional surface-to-surface contact type, with a friction coefficient of 0.2. This friction coefficient was enough to prevent any lateral slide between the sample and the compression plates. 

**Figure 7:**
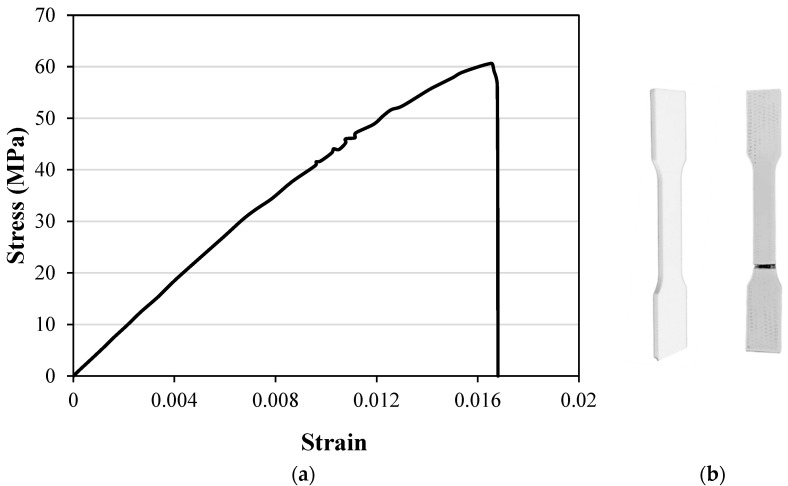
Tensile test (**a**) stress–strain curve of the PLA plastic. (**b**) Tensile test sample before and after the test.

The MAT24 piecewise linear plasticity material model was used to define the material properties of the cellular structures. The mechanical properties of PLA were obtained by conducting tensile experiments according to the ASTM D638-14 test standard [56]. The tensile samples were prepared using FDM with the same printing parameters used for re-entrant structures. Figure 7 shows the tensile stress–strain curve. The mechanical properties of PLA, as calculated from the stress–strain curve, are Young’s modulus of 4.1 GPa, an ultimate strength of 59 MPa, and Poisson’s ratio of 0.35. The PLA material has a density of 1.25 gr/cm^3^.

**Figure 8:**
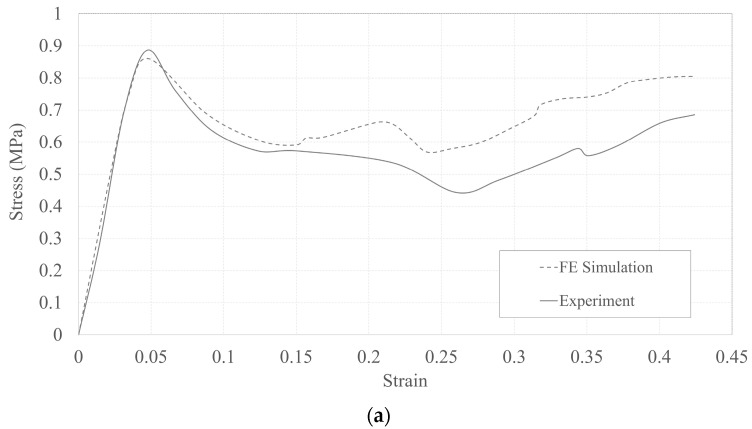

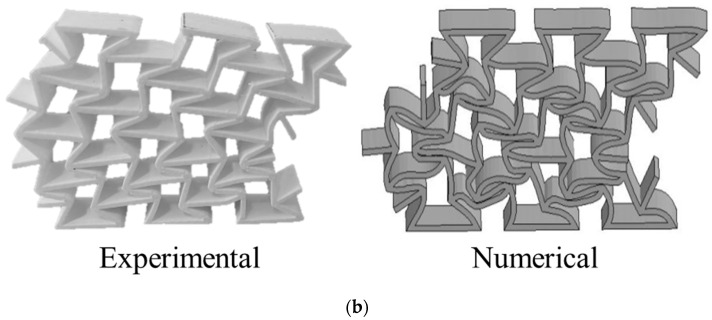
Comparison of experimental and numerical results: (**a**) force-displacement response and (**b**) deformation mode.

The model predictions were compared against experimental results to check the ability of the model to capture the required responses. A standard RH design, with $l_{cl}=l_{ds}=7\;mm$ and $t_{cl}=t=1\;mm$, was used to validate the numerical model. Figure 8 presents the numerical and experimental stress–strain responses and the deformation modes of the structure. It is clear from the stress–strain curves that the numerical model can reasonably predict the elastic and post-collapse responses of the structure. Similarly, the model was able to capture well the deformation pattern of the honeycomb. In the remainder of the paper, the FE model was mainly used to discuss the deformation modes and to estimate Poisson’s ratio in the elastic phase; therefore, its accuracy, as discussed earlier, was deemed enough for the scope of its use in the current study.

## 3. Results and Discussion

### 3.1. Stress–Strain Response and Deformation Mode

**Figure 9:**
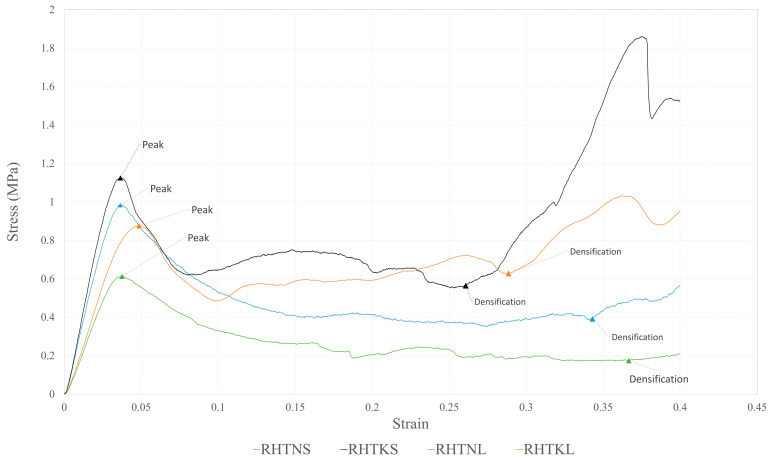
Stress–strain curves for re-entrant honeycombs with different crosslinks configurations.

The macroscopic behaviours of the re-entrant honeycombs, represented by the stress–strain curves obtained from the compression tests, are presented in Figure 9. All honeycombs were compressed to 40% of their initial length, which was enough for the structures to collapse and reach the densification stage. The figure confirms that all honeycombs follow the typical stress–strain response and show three distinctive regions, including elastic, plateau, and densification.

At the early stages of the compression process, the walls of re-entrant cells bend with small deflections, resulting in a linear elastic response. During this stage (i.e., the elastic stage), the re-entrant cells deform uniformly and the stress value increases steeply with the deformation until reaching a peak stress. It is clear from Figure 9 that the studied structures with different crosslink parameters have different slopes of the linear responses and different peak stress values. This indicates that the crosslink’s geometry influences the elastic responses of the structures. The peak stresses and the slopes of the elastic lines are more significant in the honeycombs with shorter crosslinks, i.e., RHTNS and RHTKS. 

When the peak stress is reached, localized deformation, in the form of plastic yielding (i.e., formation of plastic hinges) or brittle fracture, takes place for the cells within specific rows of the structure. The local deformation is caused by the rotation of the diagonal struts due to the formation of plastic hinges, i.e., plastic yielding, at the connecting nodes. This collapse causes the stress value to drop, indicating the start of the plateau stage. Once a layer, i.e., a row of cells, is fully deformed with the cell walls touching each other, the layer becomes stiff; therefore, the deformation propagates to adjacent layers, resulting in a progressive layer-by-layer collapse of the structure. For honeycombs with thin crosslinks, i.e., RHTNL and RHTNS, the stress levels during the plateau stage remain almost constant and show negligible fluctuations. Conversely, for RHTKL and RHTKS, the plateau stress exhibits a slight strain-hardening trend because the plateau stress increases as strain increases. As seen from the figure, the plateau stage responses of the different structures are different, which further confirms the influence of the crosslink’s configuration on the mechanical behaviour of re-entrant structures. The honeycombs with thick crosslinks, i.e., RHTKL and RHTKS, have higher plateau stresses. 

When all rows collapse, the cells’ walls begin to touch each other, indicating the start of the densification stage. This stage is associated with a steep increase in the stress response. Densification is the practical limit of the energy absorption process because the honeycomb at this strain becomes very stiff and requires a significant load to deform slightly, resulting in a limited energy absorption capability. Therefore, in energy absorption research, the energy absorption is calculated up to the densification point. Figure 9 shows that the structures with thick crosslinks (RHTKL and RHTKS) reach the densification stage earlier than their counterparts with thin crosslinks (RHTNL and RHTNS). 

**Figure 10:**
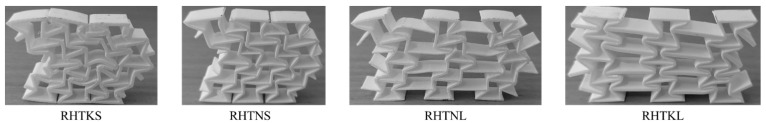
Final deformation pattern of re-entrant honeycombs with different crosslink configurations.

Figure 10 shows the final deformation modes of the compressed honeycombs. Some of the TNL crosslinks in the RHTNL structure underwent buckling deformation. No buckling was observed for other honeycombs. RHTKS and RHTNS honeycombs underwent a shifting deformation, in which the cells at the middle part of the structure moved sideways. The stiff crosslinks of these honeycombs may have caused this shifting deformation. The shifting deformation is popular for re-entrant honeycombs at high strains and generally leads to losing the global auxetic behaviour [25].

**Figure 11:**
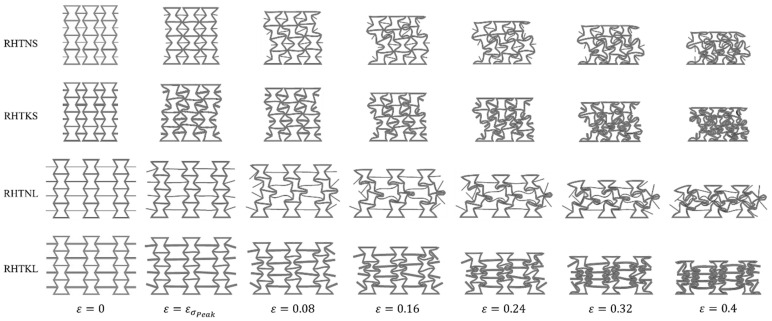
The deformation history of all investigated RH structures.

To further understand the influence of the crosslink’s parameters on the deformation behaviour, the FE model was utilized to extract the deformed profiles of RH structures at different strains, as shown in Figure 11. This figure manifests the auxetic performance of RH structures in the elastic stage, i.e., when $\varepsilon =\varepsilon_{\sigma_{Peak}}$, as all RH designs shrink in the horizontal direction due to the applied axial compression load. The numerical deformed profiles also capture the shifting deformation behaviour of the RHTNS and RHTKS structures at high deformation strains. Similarly, it shows that the RHTNL and RHTKL structures have less tendency to adopt the shifting deformation mode and somewhat maintain auxetic behaviour throughout the deformation process; this agrees well with the experimental observations, as depicted in Figure 10. 

### 3.2. Mechanical Properties

The mechanical properties, namely Young’s modulus (E), peak stress ($\sigma_{Peak}$), Poisson’s Ratio (PR), plateau stress $\left(\sigma_{Pl}\right)$, densification strain ($\varepsilon_{D}$), toughness (U), and specific energy absorption (SEA), of the different re-entrant honeycombs were extracted from the stress–strain curves discussed earlier. E and $\sigma_{Peak}$ are related to the elastic responses of the structures, representing the slope of the linear region and the maximum stress observed at the end of it, respectively. $\varepsilon_{D}$ represents the strain at which the densification stage starts. $\varepsilon_{D}$ was determined based on the energy absorption efficiency method [57]. $\sigma_{Pl}$ was taken as the average stress over the plateau stage bonded by the strain corresponding to $\sigma_{Peak}$, named as $\varepsilon_{\left(\sigma_{Peak}\right)}$, and $\varepsilon_{D}$. $\sigma_{Pl}$ can be represented mathematically as shown in Equation (1):

(1) $$\sigma_{Pl}=\frac{\int_{\varepsilon_{\left(\sigma_{Peak}\right)}}^{\varepsilon_{D}}\sigma \left(\varepsilon \right)d\varepsilon }{\varepsilon_{D}-\varepsilon_{\left(\sigma_{Peak}\right)}}$$

U is the area under the stress–strain curve up to the densification strain and it represents the energy absorbed per unit volume of the structure. U is computed using Equation (2):

(2) $$U=\int_{0}^{\varepsilon_{D}}\sigma \left(\varepsilon \right)d\varepsilon$$

SEA is the energy absorbed per unit mass of the structure and it can be calculated according to Equation (3):

(3) $$SEA=\frac{U}{\rho_{RH}}$$

where $\rho_{RH}$ is the density of the re-entrant honeycomb.

PR is the ratio between the lateral strain and axial strain of the structure. PR can be calculated using Equation (4):

(4) $$PR=-\frac{\varepsilon_{L}}{\varepsilon_{A}}$$

where $\varepsilon_{L}$ and $\varepsilon_{A}$ are the lateral and axial strains, respectively. 

The $\varepsilon_{L}$ of the lattice was taken as the average of the lateral strains of all layers. The lateral strain for each layer ($\varepsilon_{Li}$) and the overall lateral strain ($\varepsilon_{L}$) were calculated using Equations (5) and (6):

(5) $$\varepsilon_{Li}=\frac{{∆L}_{L(i)}}{L}$$

(6) $$\varepsilon_{L}=\frac{\sum_{i=1}^{i=N}\varepsilon_{Li}}{N}$$

where ${∆L}_{L(i)}$ is the dimensional change in the lateral direction for each layer. *L* is the length of the lattice. *N* is the number of layers within the RH (in this study, *N* = 4). ${∆L}_{L(i)}$ was estimated based on the deformed profiles, as captured from FE models at the peak crush force ($\varepsilon =\varepsilon_{\sigma_{Peak}}$). This is mainly due to the fact that the auxetic behaviour is normally lost at significant plastic deformation, as has been discussed earlier. 

**Figure 12:**
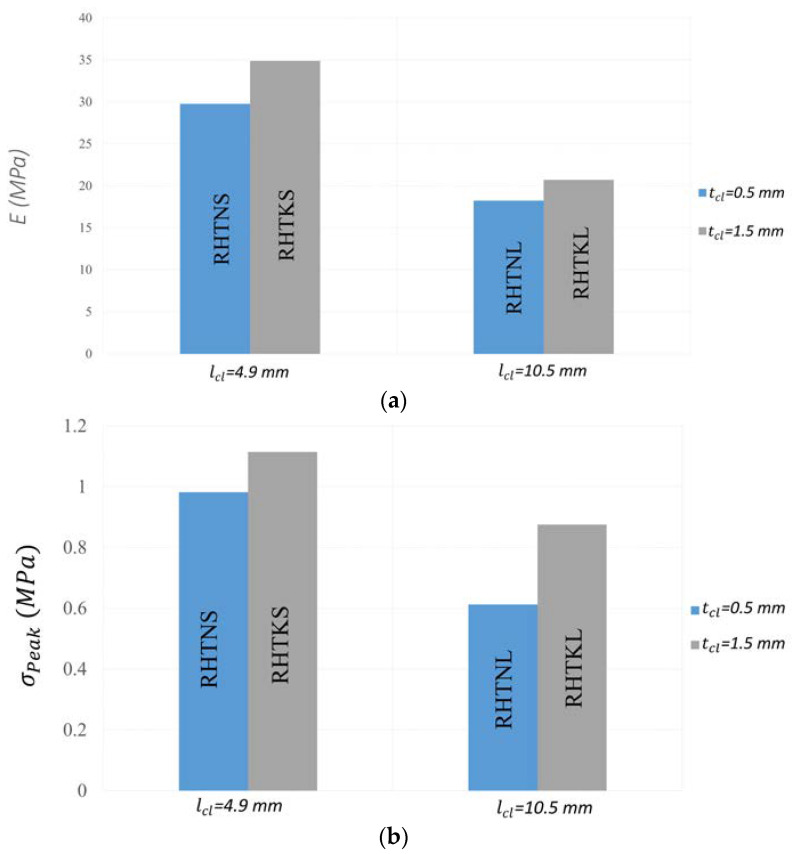

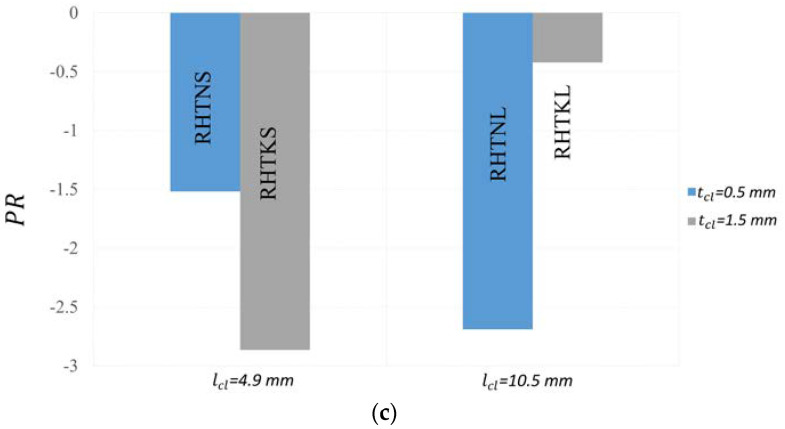
Mechanical properties of re-entrant honeycombs with different crosslink configurations (**a**) Young’s modulus. (**b**) Peak stress. (**c**) Poisson’s ratio.

The influence of the crosslink’s geometrical dimensions on Young’s modulus is depicted in Figure 12a. The honeycombs with thicker and shorter crosslinks have a higher stiffness (less elastic deformation) than the honeycombs with thinner and longer crosslinks. The influence of $l_{cl}$ on E is greater than the influence of $t_{cl}$. The maximum change in E with $l_{cl}$ is 68.25%, while the maximum change in E with $t_{cl}$ is 17.16%.

The changes in peak stresses with the length and thickness of the crosslinks are plotted in Figure 12b. Increasing the thickness or decreasing the length of the crosslinks results in increasing the peak stress. This indicates that the structures with thicker and shorter crosslinks require higher stresses to collapse plastically. The influence of $l_{cl}$ on $\sigma_{Peak}$ is greater in honeycombs with thinner crosslinks. The peak stress dropped by 21.44% and 37.58% in honeycombs with thick and thin crosslinks, respectively, when $l_{cl}$ increased from 4.9 mm to 10.5 mm.

Figure 12c displays the PRs of all RHs. RH designs with shorter crosslinks have a greater auxetic performance than those structures with longer links. These results agree well with the observed deformation patterns presented in Figure 11. This behaviour can be elucidated by the effect of the high stiffness of the shorter crosslinks. The stiff crosslinks, in RH with shorter crosslinks, induce greater bending moments in the inclined struts of the re-entrant cell, leading to a higher deflection/rotation of these struts and a greater dimensional change in the lateral direction. RHTKS and RHTNS demonstrate comparable PRs of −2.86 and −2.68, respectively. RHTKL exhibits the lowest PR of −0.55, corresponding to an average lateral displacement of −1.5 mm.

**Figure 13:**
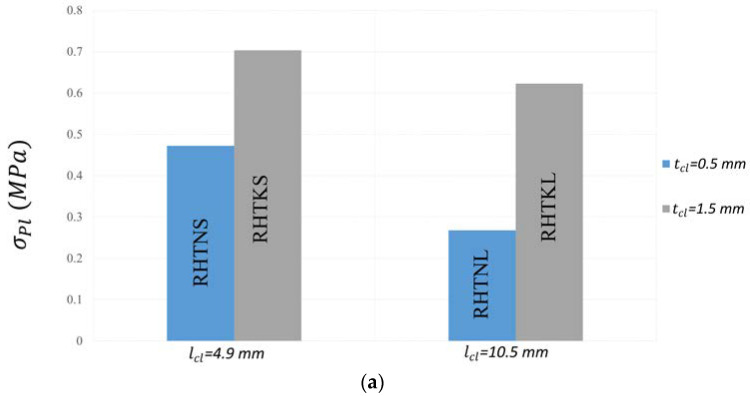

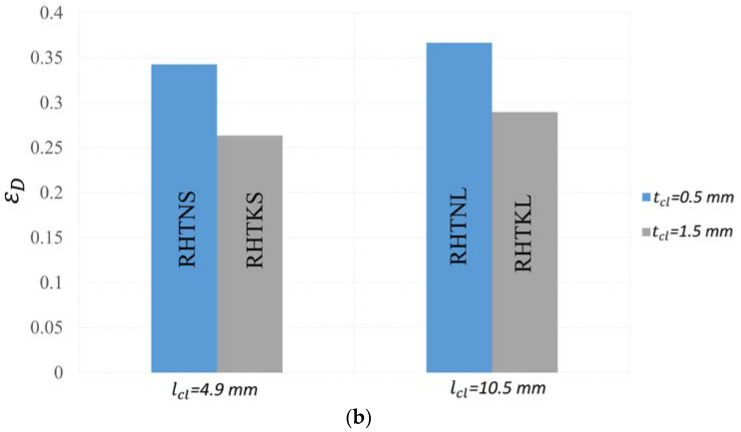
Mechanical properties of re-entrant honeycombs with different crosslink configurations (**a**) Plateau stress. (**b**) Densification strain.

Figure 13a illustrates the variations of $\sigma_{Pl}$ with the parameters of the crosslinks. Similar to $\sigma_{Peak}$, $\sigma_{Pl}$ was higher in the honeycombs with shorter and thicker crosslinks. The RHTKS honeycomb with $l_{cl}=4.9\;mm$ and $t_{cl}=1.5\;mm$ offers a plateau stress value of 0.7 MPa, which is 162% greater than that of the RHTNL honeycomb with $l_{cl}=10.5\;mm$ and $t_{cl}=0.5\;mm$. 

Figure 13b shows the values of the densification strain versus the parameters of the crosslinks. As can be observed, $\varepsilon_{D}$ strain increases by increasing the length or decreasing the thickness of the crosslinks. The influence of $t_{cl}$ on $\varepsilon_{D}$ is more profound compared to the influence of $l_{cl}$. By increasing $l_{cl}$ from 4.9 mm to 10.5 mm, the biggest change in $\varepsilon_{D}$ was around 9.88%, observed in the honeycombs with thicker crosslinks. On the other side, $\varepsilon_{D}$ changed by ~30% when changing the thickness in the honeycombs with short crosslinks.

**Figure 14:**
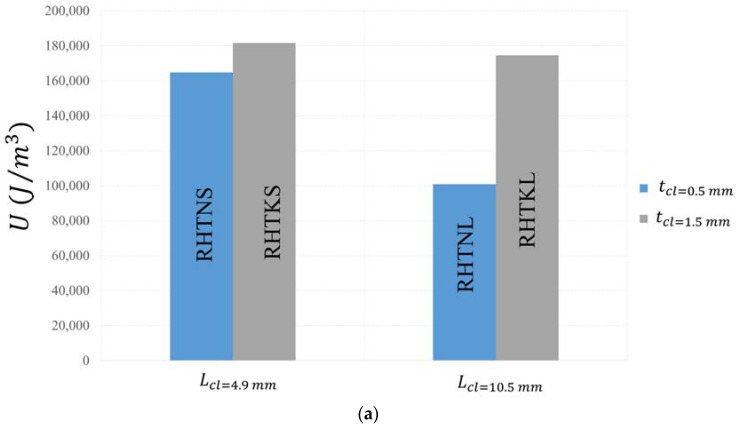

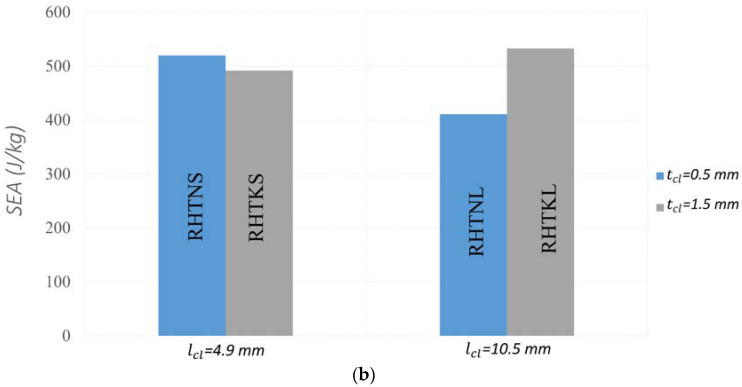
Mechanical properties of re-entrant honeycombs with different crosslink configurations (**a**) Toughness. (**b**) Specific energy absorption.

The changes of toughness with the thickness and length of crosslinks are displayed in Figure 14a. U increases with increasing thickness and decreasing length. The RHTKS honeycomb with $l_{cl}=4.9\;mm$ and $t_{cl}=1.5\;mm$ absorbed 181,644.65 J/m^3^, which is 80% greater than that of the RHTNL honeycomb with $l_{cl}=10.5\;mm$ and $t_{cl}=0.5\;mm$. U is more sensitive to $t_{cl}$ when $l_{cl}$ is bigger. Changing $t_{cl}$ from the upper limit to the lower limit reduced U by 9% and 73% in the honeycombs with short and long crosslinks, respectively. Conversely, U is more sensitive to the $l_{cl}$ in honeycombs with thinner crosslinks. U increased by 63.3% and 4% when decreasing $l_{cl}$ in honeycombs with thin and thick crosslinks, respectively.

The influence of crosslink dimensions on SEA is presented in Figure 14b. It appears that SEA has no consistent trend with the parameters of crosslinks. SEA increases with increasing $l_{cl}$ in the structures with thick crosslinks, while it decreases in the structures with thin crosslinks. Similarly, SEA decreases with $t_{cl}$ in the structures with short crosslinks, while it increases in the structures with long crosslinks. This behaviour might be because any change in the size of the crosslink yields a change in the mass of the structure and this balances out any changes in the energy absorption capacity. The RHTKL honeycomb, with $l_{cl}=10.5\;mm$ and $t_{cl}=1.5\;mm$, outperforms all other honeycombs, showing the highest specific energy absorption capacity of 532.59 J/kg.

## 4. Conclusions

Unlike previous research in the field, this paper focused mainly on the influence of the crosslink’s length and thickness on the mechanical and energy absorption properties of auxetic re-entrant honeycombs. Four different non-homogenous honeycombs with thin–long, thin–short, thick–long, and thick–short chevron crosslinks were additively manufactured using the Fused Deposition Modelling (FDM) additive manufacturing technique. Experimental quasi-static compression tests were executed to extract the crush and mechanical properties of the honeycombs. Numerical simulations were also utilized to examine the auxetic deformation patterns of the different structures. The parametric analysis of the impact of crosslinks revealed the following:

- By increasing the length of the crosslinks, the elastic modulus, peak stress, and plateau stress decrease, while the densification strains increase.
- By increasing the thickness of the crosslinks, the elastic modulus, peak stress, and plateau stress increase, while the densification strains decrease.
- Poisson’s ratio increases with a decreasing length of crosslinks.
- Toughness increases by reducing the thickness and length of the crosslinks.
- Among different honeycombs, the RHTKS honeycomb, with thick–short crosslinks, exhibited the highest elastic modulus, and peak and plateau stresses, while it showed the lowest densification strain.
- The RHTKL honeycomb, with thick–long crosslinks, absorbed the highest amount of energy per unit mass (SEA) due to its increased mass.

This study confirms that controlling the crosslink parameters is a feasible and easy way to alter the mechanical properties of the re-entrant honeycombs. As this study only focused on honeycombs with horizontal crosslinks, further research is recommended to explore the impact of the crosslinks on the honeycombs with vertical crosslinks.

## Data Availability

The original contributions presented in the study are included in the article, further inquiries can be directed to the corresponding author.
